# Regional Gray Matter Volume Identifies High Risk of Unsafe Driving in Healthy Older People

**DOI:** 10.3389/fnagi.2020.592979

**Published:** 2020-12-03

**Authors:** Yasuharu Yamamoto, Bun Yamagata, Jinichi Hirano, Ryo Ueda, Hiroshi Yoshitake, Kazuno Negishi, Mika Yamagishi, Mariko Kimura, Kei Kamiya, Motoki Shino, Masaru Mimura

**Affiliations:** ^1^Department of Neuropsychiatry, Keio University School of Medicine, Tokyo, Japan; ^2^Office of Radiation Technology, Keio University Hospital, Tokyo, Japan; ^3^Department of Human and Engineered Environmental Studies, The University of Tokyo, Tokyo, Japan; ^4^Department of Ophthalmology, Keio University School of Medicine, Tokyo, Japan; ^5^Graduate School of Psychology, Rissho University, Tokyo, Japan

**Keywords:** gray matter volume, healthy older people, machine learning, on-road driving, support vector machine, unsafe driving

## Abstract

In developed countries, the number of traffic accidents caused by older drivers is increasing. Approximately half of the older drivers who cause fatal accidents are cognitively normal. Thus, it is important to identify older drivers who are cognitively normal but at high risk of causing fatal traffic accidents. However, no standardized method for assessing the driving ability of older drivers has been established. We aimed to establish an objective assessment of driving ability and to clarify the neural basis of unsafe driving in healthy older people. We enrolled 32 healthy older individuals aged over 65 years and classified unsafe drivers using an on-road driving test. We then utilized a machine learning approach to distinguish unsafe drivers from safe drivers based on clinical features and gray matter volume data. Twenty-one participants were classified as safe drivers and 11 participants as unsafe drivers. A linear support vector machine classifier successfully distinguished unsafe drivers from safe drivers with 87.5% accuracy (sensitivity of 63.6% and specificity of 100%). Five parameters (age and gray matter volume in four cortical regions, including the left superior part of the precentral sulcus, the left sulcus intermedius primus [of Jensen], the right orbital part of the inferior frontal gyrus, and the right superior frontal sulcus), were consistently selected as features for the final classification model. Our findings indicate that the cortical regions implicated in voluntary orienting of attention, decision making, and working memory may constitute the essential neural basis of driving behavior.

## Introduction

Driving requires the integration of sensory, motor, and cognitive functions (Hird et al., 2016). Because these functions typically decline with age, older people are at an increased risk of causing fatal traffic accidents (Anstey et al., 2005). In developed countries, the number of traffic accidents caused by older drivers is increasing over time, along with the aging population. In Japan, the number of licensed drivers aged over 65 years has increased in recent years, exceeding 18 million in 2018, and the number of fatal accidents caused by older drivers has maintained an upward trend (Ichikawa et al., 2020). Although previous studies consistently showed that individuals with dementia are at increased risk of unsafe driving among the older population (Drachman and Swearer, 1993; Frittelli et al., 2009), surprisingly, a report published by the National Police Agency revealed that approximately half of the older drivers who caused fatal accidents were cognitively normal (National Police Agency, 2020). These findings suggest that detecting dementia and mild cognitive impairment (MCI) is not sufficient to prevent fatal traffic accidents caused by older drivers.

Despite the importance of identifying characteristics of older drivers who are cognitively normal but at high risk of causing fatal traffic accidents, a standardized method for assessing the driving ability of healthy older drivers has not yet been established. Although on-road driving tests are recognized as the gold standard assessment for measuring driving ability, it is not practical to perform driving tests for all older drivers because of the cost involved (Langford et al., 2004). One previous structural magnetic resonance imaging (MRI) study including both healthy older people and those with MCI reported that gray matter volume in premotor cortex was negatively correlated with the tendency to commit driving errors assessed with the Driving Behavior Questionnaire (DBQ) (Sakai et al., 2012). This finding suggests that biological markers of unsafe driving might be captured by brain MRI; however, no previous study has assessed driving ability and investigated the neural correlates of driving ability among healthy older people.

The current study had two main aims. First, we categorized participants into unsafe or safe drivers using a new sensing method for the objective evaluation of on-road driving ability of healthy older people on the basis of vehicle behavior using a data recorder and video cameras. Second, to describe the neurobiological features associated with unsafe driving, we built a classification model to distinguish unsafe from safe drivers based on gray matter volume data using a linear support vector machine (SVM) approach.

## Materials and Methods

### Participants

The present study recruited 32 healthy older individuals aged over 65 years from the local community through online advertisements at the University of Tokyo and the Musashisakai Driving School (Tokyo, Japan). All participants were diagnosed as “cognitively normal” using the Alzheimer’s Disease Neuroimaging Initiative (ADNI) diagnostic classification: (1) Mini-Mental State Examination (MMSE) score between 24 and 30; (2), Clinical Dementia Rating (CDR) score of 0; (3) normal memory function measured by education-adjusted scores on the Logical Memory II subscale of delayed paragraph recall from the Wechsler Memory Scale—Revised (WMS-R) (Petersen et al., 2010). Participants were confirmed to have had no lifetime history of diagnoses of psychiatric or neurological conditions. Participants were also required to maintain a current valid driver’s license and to still be actively driving at the time of the study. After an extensive description of the study, written informed consent was obtained from all participants prior to enrollment and investigations were performed in accordance with the ethical standards of the Declaration of Helsinki. The study protocol was approved by the ethics committees of the University of Tokyo and Keio University. After all participants took an on-road driving test at the Musashisakai Driving School, they were moved to Keio University Hospital and underwent cognitive assessments, a visual function test, and an MRI scan.

### Measurements

#### Cognitive Assessment

The general cognitive function of each participant was assessed using the Raven’s Colored Progressive Matrices (RCPM). The Rey Auditory Verbal Learning Test (RAVLT), the Rey–Osterrieth Complex Figure Test (ROCFT), the Clock Drawing Test (CDT), and the Everyday Memory Checklist (EMC) were used to evaluate memory and visuospatial function. We estimated attentional/executive function using the Stroop Test (ST), the Trail Making Test (TMT) A and B, and the Dysexecutive Questionnaire (DEX). We investigated subjective driving ability using the DBQ. Depression severity was evaluated using the Geriatric Depression Scale (GDS). Handedness was assessed using the Edinburgh Handedness Inventory (Oldfield, 1971). The CDT was scored using a five-point scoring system adopted from the ADNI’s cognitive assessments (Iwatsubo et al., 2018). Clinical neuropsychologists (MY and KK) administered all cognitive assessments in an environment with adequate lighting and reduced noise conditions.

#### Cambridge Neuropsychological Test Automated Battery

In addition to these neuropsychological tests mentioned above, we performed the Cambridge Neuropsychological Test Automated Battery (CANTAB), which is a computer-based test battery widely used in neurocognitive studies (Cambridge Cognition, Cambridge, United Kingdom) (Robbins et al., 1994). Specifically, we adopted the CANTAB battery consisting of four cognitive domain tasks to assess subtle cognitive changes with aging (Soares et al., 2015): (1) visual memory (paired associates learning [PAL]); (2) attention (reaction time [RTI]); (3) working memory (spatial working memory [SWM]); (4) control task measuring simple psychomotor speed and accuracy (motor screening task [MOT]). According to the standard protocol, the instructions for the tests were explained to the participants before initiation of the study. The standard instructions for the tests were provided in the CANTAB manual and were translated into Japanese. The execution of the tasks required approximately 20 min. The test battery was administered in a silent room without distractions. Details of the procedures are available elsewhere (Akter et al., 2015).

#### Functional Visual Acuity Test

We adopted binocular functional visual acuity to assess the visual function associated with driving ability. First, we measured corrected distance visual acuity (CDVA) using Landolt vision charts. Second, we calculated corrected distance functional visual acuity (CDFVA) with the AS-28 FVA Measurement System (Kowa, Aichi, Japan) (Katada et al., 2016; Negishi et al., 2016). CDFVA consists of five indicators: functional visual acuity (FVA), maximum visual acuity (MaxVA), minimal visual acuity (MinVA), visual maintenance ratio (VMR), and average response time (ART). FVA, MaxVA, and MinVA represent the average, maximum, and minimum visual acuity in a period of 60 s, respectively. VMR was defined as the ratio of FVA to CDVA. ART was computed as the average response time in giving the direction of a Landolt ring, which was shown on the screen every 2 s.

#### Image Acquisition and Preprocessing

MR images were acquired using a 3.0-T MRI scanner (MAGNETOM Verio, Siemens Healthineers, Erlangen, Germany) with an 8-channel head coil. High-resolution T1-weighted images were acquired using a magnetization-prepared rapid acquisition with gradient echo sequence (repetition time: 1.9 s; echo time: 2.99 ms; flip angle: 9°; field of view: 256 mm; matrix size: 256 × 256; slice thickness: 1.2 mm; 192 sagittal slices; voxel size: 1 × 1 × 1.2 mm). All images were first visually checked for scanner artifacts and anatomical anomalies.

Structural MRI data were preprocessed using FreeSurfer’s *recon-all* processing pipeline for cortical reconstruction and volumetric segmentation (Fischl and Dale, 2000; Fischl et al., 2004) (software freely available at http://surfer.nmr.mgh.harvard.edu/). The cortical processing stream in FreeSurfer included Talairach transformation, removal of non-brain tissue, segmentation of subcortical white matter and gray matter tissue, intensity normalization and atlas registration. After these automatic steps, a triangular mesh model of the cortical surface consisting of over 150,000 vertices per hemisphere was generated, and the cortical surface was parcellated into 74 distinct cortical regions of interest (ROIs) based on curvature values of the surface for each hemisphere according to the Destrieux atlas (Destrieux et al., 2010). Each preprocessed image was visually inspected and any segmentation errors were manually corrected by a researcher. Gray matter volume for each ROI was then calculated automatically using FreeSurfer’s recon-all processing pipeline. Furthermore, ROI gray matter volumes were divided by each subject’s estimated total intracranial volume (eTIV) to adjust for individual differences in overall cranial size (O’Brien et al., 2011).

#### On-Road Driving Test

**FIGURE 1:**
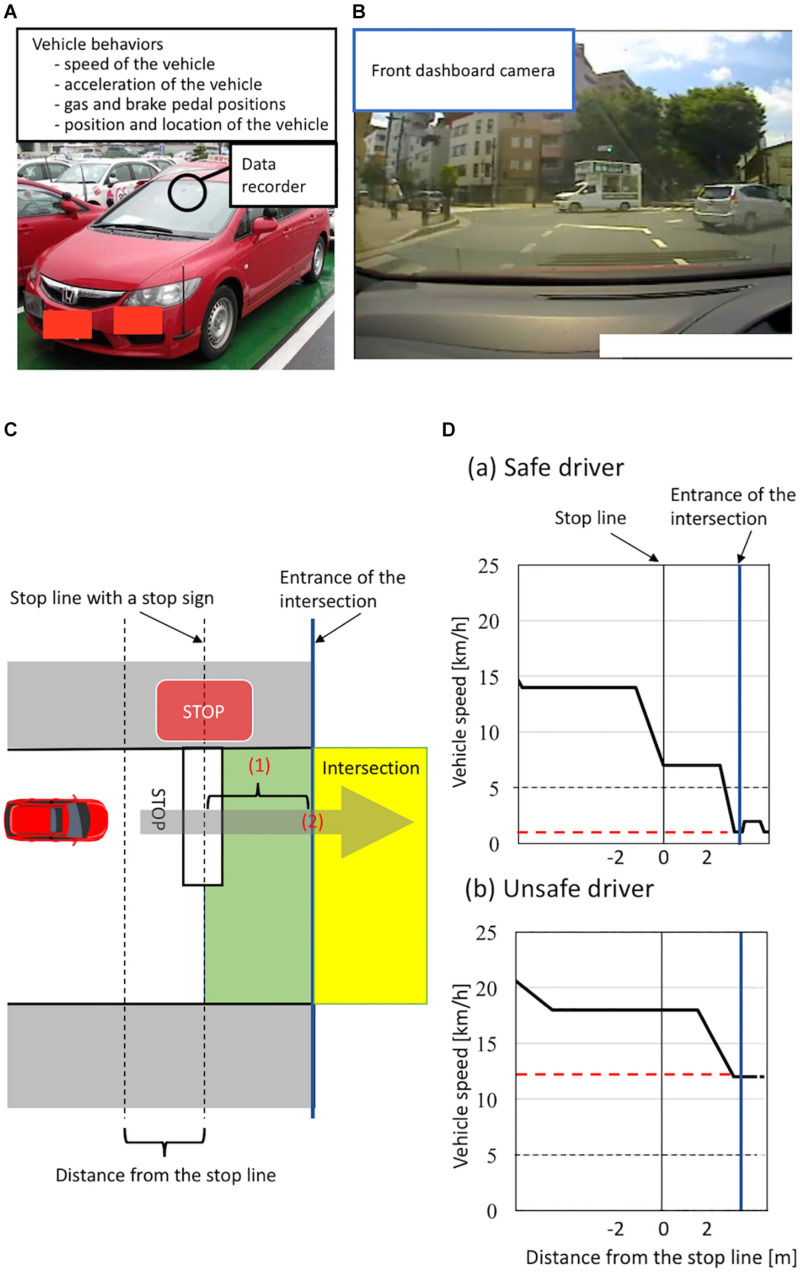
**(A–B)** Instrumented automatic vehicle for evaluating vehicle behaviors using a data recorder (Tough More-eye S manufactured by Finefit Design, Aichi, Japan) and charge-coupled device (CCD) cameras. **(A)** The data recorder provides the speed and acceleration of the vehicle and the positions of gas and brake pedal. The data recorder was placed on the front dashboard. **(B)** The front dashboard CCD camera filmed surrounding traffic and lanes. **(C–D)** Classification of a safe or unsafe driver based on vehicle behaviors. **(C)** The definition of the area of the intersection having a stop sign. Vehicles are driven on the left side of the road in Japan. The area from the stop line to the entrance of the intersection is shown in green. The area of the intersection is shown in yellow. The entrance of the intersection is shown by a blue line. To evaluate drivers’ behavior, we measured (1) the minimum speed of the vehicle between the stop line and the entrance of the intersection and (2) the speed of the vehicle when the front of the car was at the entrance of the intersection. **(D)** Schema of velocity distribution patterns measured using the instrumented vehicle. Figures show examples of velocity distribution patterns of (a) a safe driver and (b) an unsafe driver. The minimum speed of a vehicle from the stop line to the entrance of the intersection is shown by a red line. We classified all participants into two groups according to (1) whether the minimum speed of the vehicle between the stop line and the entrance of the intersection was less than 5 km/h and (2) whether the speed of the vehicle when the front of the car was at the entrance of the intersection was less than 5 km/h. We classified participants who met both criteria as safe drivers, and classified those who did not as unsafe drivers.

To evaluate the on-road driving ability of healthy older individuals on the basis of vehicle behaviors, we used an instrumented automatic vehicle with a data recorder, charge-coupled device (CCD) cameras, and dual brake controls (Figures 1A,B; Shino et al., 2018). The data recorder (Tough More-eye S manufactured by Finefit Design, Aichi, Japan) provides vehicle information, including speed, acceleration, and gas and brake pedal positions. CCD cameras filmed the driver’s face, gaze, and footwork and surrounding traffic and lanes. The position and location of the vehicle was determined using data from these cameras. On-road driving tests were performed at the Musashisakai Driving School in suburban Tokyo. All participants drove the instrumented vehicle on the same course in a city area around the driving school for 30 min. To ensure participant safety, a driving instructor accompanied participants in the car while they drove the vehicle.

#### Classification of Safe and Unsafe Drivers

We evaluated participants’ driving ability at intersections with a stop sign using on-road driving test data, because older drivers most frequently cause traffic accidents at intersections without traffic lights (Cicchino and McCartt, 2015; Lombardi et al., 2017). At intersections with a stop sign, drivers generally need to notice the stop sign, slow down the vehicle sufficiently, then pass through the intersection without inappropriate acceleration. More precisely, drivers must decelerate the vehicle from the stop line to the entrance of the intersection sufficiently to be able to stop immediately if there are other cars or pedestrians at the intersection, and should not accelerate at the entrance of the intersection. We thus evaluated these driving behaviors at intersections using two parameters: the minimum speed of a vehicle moving past the stop line to the entrance of the intersection, and the speed of the vehicle at the entrance of the intersection (see details in Figure 1C). Specifically, we classified all participants into unsafe and safe drivers according to two criteria: (1) whether the minimum speed of the vehicle between the stop line and the entrance of the intersection was less than 5 km/h; (2) whether the speed of the vehicle was less than 5 km/h when the front of the car was at the entrance of the intersection. We classified participants who met both criteria as safe drivers, while we classified those who did not as unsafe drivers (see details in Figure 1D). We used a cut-off value of 5 km/h to divide participants into unsafe and safe drivers because automatic vehicles are designed to move forward at a speed of less than 5 km/h when drivers release the brake pedal without depressing the gas pedal (Nesamani and Subramanian, 2006; Seers et al., 2015). A previous study analyzing more than 8,000 traffic accidents reported that no serious accidents occurred when the speed of the vehicle was below 5 km/h (Kröyer, 2015). Further, we adopted the same cut-off value (5 km/h) in our previous study assessing the driving ability of older people based on the speed of the vehicle at intersections (Shino et al., 2018; Yamamoto et al., 2020). Therefore, in the present study, participants who drove a vehicle below 5 km/h at the intersection were classified as safe drivers.

#### Classification Using Machine Learning

The purpose of the current study was to build a classification model to dissociate unsafe drivers from safe drivers using a machine learning. Based on a previous study suggesting that combining neuroimaging and clinical data could improve the accuracy of predicting cognitive decline (Lahmiri and Shmuel, 2019), we made a classification model using both gray matter volume data and clinical measures. A total of 56 clinical features and a total of 148 parcellated cortical regions were included for the classification model. Further, before we created the classification model, we attempted to overcome the issue of a small sample size for classification by selecting features used in the classification model with the least absolute shrinkage and selection operator (LASSO) algorithm. In general, the LASSO algorithm performed linear regression with L1-regularization and conducted feature selection based on the regularization parameter α. In the current study, the optimal value of the regularization parameter α was achieved using the Akaike Information Criterion, which is widely used as a penalized likelihood criterion (Congdon, 2010).

In the present study, a linear SVM was used to create the classification model. The linear SVM method is widely used as a powerful supervised learning methodology in binary classification (Cortes and Vapnik, 1995). In general, linear SVM classifiers separate two groups based on the value of penalty coefficient C, which determines the learning algorithm for classification. In the current study, the optimal value of the penalty coefficient C was tuned using Optuna, which is a hyperparameter optimization framework applicable to machine learning (Akiba et al., 2019).

After fixing C for the linear SVM classifier, we evaluated the performance of the linear SVM classifier using leave-one-out cross-validation (LOOCV), which is a widely used validation method for accurately assessing the performance of predictive models. Specifically, LOOCV maximizes the training sample and avoids possible case partition bias, even with small sample sizes (Lopes et al., 2019). In our study, LOOCV continued for 32 rounds to test all samples one by one. At each round of LOOCV, one participant was selected as testing data, and the remaining 31 participants were used to train the linear SVM classifier. After 32 rounds, the accuracy, sensitivity, and specificity of the linear SVM classifier were estimated.

We used scikit-learn in Python 3.7.0 for machine-learning analyses (Pedregosa et al., 2011).

### Statistical Analysis

For clinical data, we adopted a two-tailed *t*-test, chi-squared test, or multivariate analysis of variance in the group comparison between safe drivers and unsafe drivers. IBM SPSS software Statistics 25 for Mac OS (IBM, Armonk, NY) was used for the statistical analysis. We used a liberal statistical threshold of *P* < 0.05.

## Results

### Demographic Characteristics, Neuropsychological, and Functional Visual Acuity Tests

We classified 21 participants as safe drivers and 11 participants as unsafe drivers (Table 1). There were significant differences between groups in the DEX, EMC, PAL total errors (six shapes, adjusted), and RTI simple accuracy scores. There were no differences in the sex ratio, age, duration of education, driving experience, handedness, and results of functional visual acuity test between the groups.

**TABLE 1:** Demographics and results of neuropsychological and functional visual acuity test.

Demographic data					
		Safe drivers	Unsafe drivers	*F* or *T*	*P*-value
n		21	11		
Sex male/female		20/1	10/1		0.631
Age (years)		74.9 ± 3.7	77.9 ± 4.1	2.02	0.052
Handedness		89.4 ± 34.3	100 ± 0.0	0.99	0.330
Education (years)		14.4 ± 2.1	14.5 ± 1.9	0.10	0.925
Driving experience (years)		51.0 ± 6.9	47.0 ± 14.5	0.83	0.424

**Neuropsychological tests**					

	**Subscale**	**Safe drivers**	**Unsafe drivers**	***F* or *T***	***P*-value**

MMSE total		27.5 ± 2.2	27.8 ± 1.5	0.38	0.708
Logical memory of the WMS-R				0.54	0.586
	Immediate recall	19.4 ± 5.1	17.2 ± 7.4		
	Delayed recall	15.1 ± 5.4	12.8 ± 6.2		
RCPM		29.3 ± 2.9	31.0 ± 2.9	1.53	0.135
RAVLT				1.58	0.183
	Immediate recall, 1st trial	5.2 ± 1.8	4.2 ± 1.5		
	Immediate recall, 2nd trial	7.2 ± 1.9	7.2 ± 2.0		
	Immediate recall, 3rd trial	8.8 ± 2.4	8.5 ± 1.8		
	Immediate recall, 4th trial	9.8 ± 2.6	10.0 ± 2.0		
	Immediate recall, 5th trial	10.8 ± 2.3	10.5 ± 2.3		
	Interference	4.6 ± 1.5	4.3 ± 1.8		
	Delayed recall	8.8 ± 3.1	6.7 ± 3.5		
	Recognition correct	14.0 ± 0.9	13.2 ± 3.9		
	Recognition false positive	1.1 ± 1.8	0.6 ± 1.1		
	Recognition false negative	1.0 ± 0.9	1.8 ± 3.9		
ROCFT				1.98	0.156
	Copy	35.0 ± 1.3	35.5 ± 0.8		
	Delayed recall	20.0 ± 5.0	23.9 ± 5.0		
ST	Completion time			0.64	0.598
	Part I (s)	17.1 ± 2.7	18.1 ± 3.8		
	Part II (s)	20.0 ± 3.8	21.8 ± 4.6		
	Part III (s)	28.8 ± 11.2	29.3 ± 6.3		
	Numbers of errors			0.32	0.813
	Part I	0.1 ± 0.3	0.1 ± 0.3		
	Part II	0.2 ± 0.5	0.3 ± 0.4		
	Part III	1.2 ± 1.4	0.7 ± 1.1		
TMT				0.55	0.585
	A	100.7 ± 33.9	97.3 ± 20.2		
	B	158.9 ± 79.2	133.8 ± 55.9		
CDT				0.77	0.388
	Copy	5.0 ± 0.0	5.0 ± 0.0		
	Free-drawn	4.9 ± 0.3	4.7 ± 0.4		
DEX*		10.5 ± 7.3	17.2 ± 9.4	2.15	0.040
EMC*		6.7 ± 3.8	10.8 ± 3.8	2.82	0.008
DBQ		69.5 ± 14.5	73.2 ± 15.2	0.65	0.518
GDS		1.3 ± 1.5	2.5 ± 2.3	1.68	0.104

**Cambridge Neuropsychological Test Automated Battery**					

	**Subscale**	**Safe drivers**	**Unsafe drivers**	***F* or *T***	***P*-value**

MOT					
	Latency			1.64	0.211
	Mean	799.0 ± 122.3	902.4 ± 275.1		
	Median	780.1 ± 132.8	807.2 ± 143.0		
	Mean error	10.6 ± 2.8	8.7 ± 2.3	1.85	0.075
PAL					
	TE (adjusted)	36.5 ± 23.9	32.4 ± 18.9	0.49	0.631
	TE (six shapes, adjusted)*	9.5 ± 7.6	3.6 ± 3.3	2.35	0.025
RTI					
	Simple				
	Accuracy score*	8.7 ± 0.5	9.0 ± 0.0	2.34	0.030
	Reaction time			1.41	0.261
	Mean	307.4 ± 46.5	284.1 ± 23.6		
	Median	293.4 ± 40.5	278.2 ± 25.6		
	SD	53.5 ± 32.9	31.5 ± 9.6		
	Movement time			1.06	0.382
	Mean	411.2 ± 119.9	408.1 ± 66.7		
	Median	403.6 ± 118.1	401.5 ± 63.8		
	SD	51.4 ± 25.9	37.5 ± 12.3		
	5 Choice				
	Accuracy score	7.9 ± 0.3	7.9 ± 0.3	0.04	0.969
	Reaction time			0.20	0.896
	Mean	342.1 ± 33.7	347.9 ± 45.5		
	Median	337.4 ± 36.2	345.5 ± 39.3		
	SD	43.3 ± 19.5	43.5 ± 20.0		
	Movement time			0.37	0.773
	Mean	431.9 ± 105.7	404.9 ± 73.2		
	Median	432.4 ± 106.4	401.7 ± 79.0		
	SD	39.0 ± 14.6	40.3 ± 30.1		
SWM					
	Between errors	48.3 ± 16.9	43.7 ± 11.0	0.79	0.434
	Strategy	36.8 ± 4.0	36.0 ± 2.1	0.61	0.546

**Functional visual acuity test**					

		**Safe drivers**	**Unsafe drivers**	***F* or *T***	***P*-value**

FVA (logMAR)		0.123 ± 0.132	0.216 ± 0.148	1.77	0.088
MaxVA (logMAR)		−0.019 ± 0.123	0.066 ± 0.104	1.90	0.067
MinVA (logMAR)		0.300 ± 0.217	0.385 ± 0.227	1.01	0.318
VMR		0.93 ± 0.06	0.92 ± 0.08	0.45	0.662
ART		1.44 ± 0.11	1.41 ± 0.08	0.67	0.511

*MMSE, Mini-Mental State Examination; WMS-R, Logical Memory II subscale of the Wechsler Memory Scale—Revised; RCPM, Raven’s Colored Progressive Matrices; RAVLT, Rey Auditory Verbal Learning Test; ROCFT, Rey–Osterrieth Complex Figure Test; ST, Stroop Test; TMT, Trail Making Test; CDT, Clock Drawing Test; DEX, Dysexecutive Questionnaire; EMC, Everyday Memory Checklist; DBQ, Driving Behavior Questionnaire; GDS, Geriatric Depression Scale; MOT, motor screening task; PAL TE, paired associates learning total error; RTI, reaction time; SWM, spatial working memory; FVA, Functional visual acuity; MaxVA, Maximal functional visual acuity; MinVA, Minimal functional visual acuity; VMR, Visual maintenance ratio; ART, Average response time; logMAR, Logarithm of the minimum angular resolution; SD, standard deviation. Asterisks indicate statistical significance between groups (P < 0.05).*

### Linear SVM Classifier for Unsafe Driving Using Gray Matter Volume and Clinical Features

**FIGURE 2:**
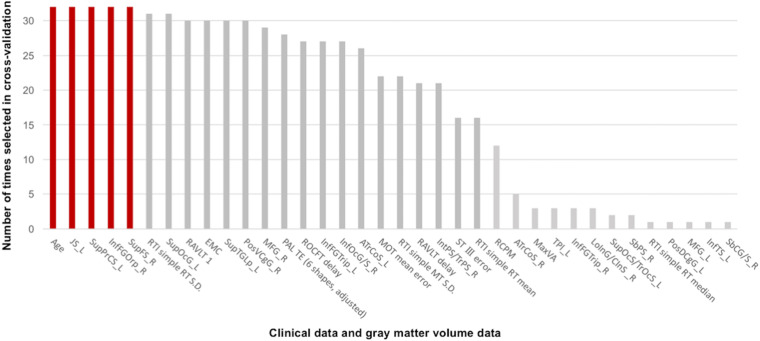
Contributions of clinical data and gray matter volume data to the classification of safe and unsafe drivers in the final model. The number of times each parameter was selected in the cross-validation is shown for all 36 parameters. Higher numbers represent a greater contribution to the classifier. Five parameters (age and gray matter volume of four cortical regions, including the left superior part of the precentral sulcus, the left sulcus intermedius primus [of Jensen], the right orbital part of the inferior frontal gyrus, and the right superior frontal sulcus) were consistently selected at every iteration. JS_L, the left sulcus intermedius primus (of Jensen); SupPrCS_L, the left superior part of the precentral sulcus; InfFGOrp_R, the right orbital part of the inferior frontal gyrus; SupFS_R, the right superior frontal sulcus; RTI, reaction time; SupOcG_L, the left superior occipital gyrus; RAVLT 1, the first trial of the Rey Auditory Verbal Learning Test immediate recall; EMC, Everyday Memory Checklist; SupTGLp_L, the left lateral aspect of the superior temporal gyrus; PosVCgG_R, the right posterior-ventral part of the cingulate gyrus; MFG_R, the right middle frontal gyrus; PAL TE, paired associates learning total error; ROCFT delay, Delayed recall of the Rey–Osterrieth Complex Figure Test; InfFGTrip_L, the left triangular part of the inferior frontal gyrus; InfOcG/S_R, the right inferior occipital gyrus and sulcus; ATrCoS_L, the left anterior transverse collateral sulcus; MOT, motor screening task; RAVLT delay, Delayed recall in the Rey Auditory Verbal Learning Test; IntPS/TrPS_R, the right intraparietal sulcus (interparietal sulcus) and transverse parietal sulci; ST III, Time taken to finish the Stroop Test part III; RCPM, Raven’s Colored Progressive Matrices; ATrCoS_R, the right anterior transverse collateral sulcus; MaxVA, Maximal functional visual acuity; TPl_L, the left temporal plane of the superior temporal gyrus; InfFGTrip_R, the right triangular part of the inferior frontal gyrus; LoInG/CInS_R, the right long insular gyrus and central insular sulcus; SupOcS/TrOcS_L, the left superior occipital sulcus and transverse occipital sulcus; SbPS_R, the right subparietal sulcus; PosDCgG_L, the left posterior-dorsal part of the cingulate gyrus; MFG_L, the left middle frontal gyrus; InfTS_L, the left inferior temporal sulcus; SbCG/S_R, the right subcentral gyrus (central operculum) and sulci.

**FIGURE 3:**
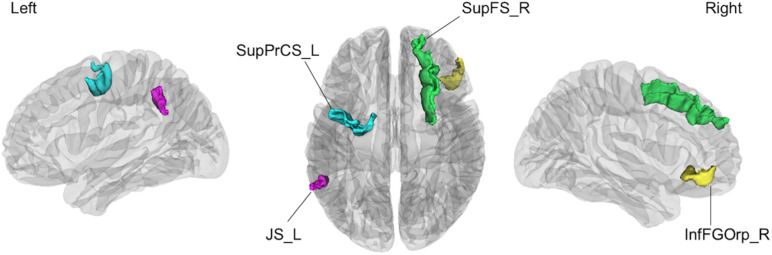
The four cortical regions identified as consistent classification inputs were located within the cortical regions involved in cognitive functions essential for driving, such as voluntary orienting of attention, decision making, and working memory. SupPrCS_L, the left superior part of the precentral sulcus; JS_L, the left sulcus intermedius primus (of Jensen); InfFGOrp_R, the right orbital part of the inferior frontal gyrus; SupFS_R, the right superior frontal sulcus.

The linear SVM classifier (α = 0.043, C = 0.027) using clinical features and gray matter volume data distinguished unsafe drivers from safe drivers with an accuracy of 87.5% (sensitivity of 63.6% and specificity of 100%). While 36 parameters were selected as features for the final classification model at least once throughout cross-validation procedures (Figure 2), five parameters (age and gray matter volume of four cortical regions, including the left superior part of the precentral sulcus, the left sulcus intermedius primus [of Jensen], the right orbital part of the inferior frontal gyrus, and the right superior frontal sulcus) were consistently selected at every iteration (Figure 3). In an additional analysis, when we selected only these five parameters as input data for classification, the linear SVM classifier (*C* = 0.050) successfully differentiated unsafe drivers from safe drivers with accuracy of 87.5% (sensitivity of 81.8% and specificity of 90.5%).

## Discussion

In the present study, the linear SVM classifier using both clinical features and gray matter volume data differentiated unsafe drivers from safe drivers with an accuracy of 87.5% (sensitivity of 63.6%, and specificity of 100%). Furthermore, in the final classification model, age and gray matter volume in four cortical regions, including the left superior part of the precentral sulcus, the left sulcus intermedius primus (of Jensen), the right orbital part of the inferior frontal gyrus, and the right superior frontal sulcus, were selected as consistent features, suggesting that regional gray matter volume changes in these four cortical regions are strongly associated with a high risk of unsafe driving among healthy older people.

One advantage of the present study is that we objectively evaluated on-road driving behaviors. In previous structural MRI studies investigating the neural basis of driving ability among older people, interviews or questionnaires were often utilized for driving evaluation (Sakai et al., 2012; Park et al., 2013; Jang et al., 2018). Furthermore, even in standardized on-road driving tests, such as Iowa’s driving test, scores are provided by a driving instructor (Dawson et al., 2009). We therefore consider that these measurements may not accurately estimate a participant’s driving ability because the actual vehicle’s behaviors on the road were not assessed. To overcome these methodological issues, we assessed driving ability according to vehicle behaviors during on-road driving test using an instrumented automatic vehicle with a data recorder and CCD cameras (Shino et al., 2018; Yamamoto et al., 2020). Specifically, focusing on driving behaviors at intersections, we evaluated participants’ driving ability using the actual speed of the vehicle at intersections, because older drivers most frequently cause traffic accidents at intersections (Cicchino and McCartt, 2015; Lombardi et al., 2017).

Our final classification model successfully dissociated unsafe drivers from safe drivers with 87.5% accuracy. We consider that the accuracy of our model was relatively high, because in two previous studies using only neuropsychological tests to predict driving ability, prediction accuracies were 66 and 90%, respectively (Brown et al., 2005; Ott et al., 2008). Furthermore, our final model identified age and gray matter volume of four cortical regions as consistent features for classification. In an additional analysis using only these five features, our model successfully dissociated unsafe drivers from safe drivers with 87.5% accuracy. This finding further suggested that the driving ability of healthy older people could be predicted accurately using only information about age and MRI data. Given that both age and MRI data are rater-independent variables, our classification model appears to be reliable, with a range of potential clinical applications.

The current results revealed that the risk of unsafe driving increases with age. A recent meta-analysis of global longitudinal cohort data revealed that all cognitive domains, particularly attentional function, decline with age (Lipnicki et al., 2017). Driving ability is also reported to be affected by aging. For example, a 2-year longitudinal study that observed the change in driving ability in older people described a gradual decline in driving ability (Duchek et al., 2003). Similarly, a recent large-sample study among older people concluded that age was the most consistent predictor of on-road driving ability (Anstey et al., 2017). However, because all of these previous studies included both healthy people and people with MCI in their analyses, the effect of age on driving ability in healthy older people was not evident. The present results therefore expand on prior findings to the extent that even in cognitively normal older people, there is a strong relationship between unsafe driving and aging.

The current data revealed that regional gray matter volume changes are highly predictive of driving ability in healthy older people. This finding suggests that gray matter volume accurately reflects changes in cortical structure related to decreased driving ability among healthy older people. One previous study examining the association of cortical changes with driving ability among older people reported that gray matter volume was correlated with driving ability, supporting the current results (Sakai et al., 2012). In the present study, four cortical regions (the left superior part of the precentral sulcus, the left sulcus intermedius primus [of Jensen], the right orbital part of the inferior frontal gyrus, and the right superior frontal sulcus) were identified as consistent classification inputs to dissociate unsafe drivers from safe drivers using the linear SVM.

The superior part of the precentral sulcus is located in the dorsal premotor cortex (PMC) including the frontal eye field (FEF). The FEF plays a decisive role in saccade programming and shows enhanced responses to a visual stimulus when it is the saccade target (Ptak and Schnider, 2010). The sulcus intermedius primus (of Jensen) is located in the inferior parietal lobule (IPL), including the supramarginal and angular gyri, which is involved in visual attention or motion perception (Zhang and Li, 2014). Importantly, the dorsal attention network (DAN) linking the FEF with the IPL is involved in voluntary orienting of visuospatial attention (Ptak, 2012; Tamber-Rosenau et al., 2018). Furthermore, the DAN improves target detection and behavioral performance by activating the visual cortex prior to the appearance of the target, particularly during anticipatory attention, in which advanced information is utilized to orient visuospatial attention to the location of an impending target, such as a road sign (Bressler et al., 2008). The DAN thus plays a key role in a goal-directed control of perceptual processing (i.e., top-down attention) (Meehan et al., 2017). In contrast, the ventral attention network (VAN) is engaged in the detection of salient and unexpected events (Corbetta and Shulman, 2002). The VAN redirects attention from the present focus to the novel stimulus of interest when very important or noticeable events are detected outside of the present focus of attention, such as a sudden pedestrian crossing, and the VAN is thus considered to be involved in bottom-up attention (Long and Kuhl, 2018). Regarding older drivers, top-down attention has been shown to compensate for reduced road hazard detection due to age-related bottom-up attentional decline, and diminished top-down attention has been shown to lead to vehicle accidents caused by older drivers (Feng et al., 2018).

The right orbital part of the inferior frontal gyrus plays a crucial role in decision making (Besnard et al., 2017; Vaidya and Fellows, 2020). A previous longitudinal neuroimaging study reported that less thinning of the orbitofrontal cortex during adolescence is associated with risky driving behavior in young people (Vijayakumar et al., 2019). Given that a previous driving simulator study reported that the number of violations and accidents was positively correlated with the tendency to make risky decisions in dilemma situations (Ba et al., 2016), structural alterations of this cortical region may have strong effects on unsafe driving behaviors.

The superior frontal sulcus has been repeatedly shown to contribute to working memory in functional MRI studies using the N-back task (Carlson et al., 1998; Heinzel et al., 2016). In our previous study examining the associations between neuropsychological tests and driving ability in healthy older people, lower working memory function was associated with greater risk of unsafe driving (Yamamoto et al., 2020). Furthermore, working memory has been reported to be associated with a driver’s ability to retain traffic information for several seconds (Da-Wei et al., 2017) and predict traffic conditions (Jipp and Ackerman, 2016). Considering that our final model identified the cortical regions involved in cognitive functions essential for driving, such as voluntary orienting of attention, decision making, and working memory, as important inputs, the current study provides new insights into the neural basis of driving behavior.

### Limitation and Future Works

The results of the current study should be interpreted with caution because of several limitations. First, the number of participants per group was relatively small. In general, small numbers of participants can induce over-fitting. To mitigate this problem, we created a sparse model using the LASSO algorithm. However, it would be optimal to train our classification model with an independent cohort to generalize the model. Future studies with larger samples at multiple sites may be useful for addressing this issue. Second, although we measured on-road driving ability using a data recorder and CCD cameras, we only evaluated one aspect of driving ability. Because we decided not to use other data, including data regarding the pedal position, gaze and footwork, to simplify the classification criteria, it may be valuable to establish a new objective method with which to measure various types of driving ability. For instance, examining driving behaviors when turning right at an intersection or making a lane change could provide useful results. Third, we used a cut-off value of 5 km/h to divide participants into unsafe and safe drivers based on the past findings (Nesamani and Subramanian, 2006; Seers et al., 2015). However, even though using such criteria for the classification, we are not able to completely eliminate the possibility of arbitrariness. Therefore, future studies with large samples are needed to confirm the validity of our classification of safe and unsafe drivers. Finally, previous studies reported that motor dysfunction (Anstey et al., 2017) and hearing impairment (Edwards et al., 2016) are associated with unsafe driving. However, motor and hearing functions were not systematically evaluated in the current study, although the participants were apparently free from these problems. Future studies should be conducted to evaluate motor and hearing functions in more detail.

## Conclusion

Overall, we built a reliable classification model for identifying the on-road driving ability of healthy older individuals with an accuracy of 87.5%. Five parameters (age and gray matter volume in four cortical regions, including the left superior part of the precentral sulcus, the left sulcus intermedius primus [of Jensen], the right orbital part of the inferior frontal gyrus, and the right superior frontal sulcus), were consistently selected as features for the final classification model. Importantly, the current findings revealed the neural bases of unsafe driving in healthy older people, suggesting that age and gray matter volume data can provide useful information for identifying unsafe drivers, potentially leading to the development of new interventions to prevent fatal traffic accidents.

## Data Availability Statement

The data that support the findings of this study are available on request to the corresponding author.

## Ethics Statement

The studies involving human participants were reviewed and approved by the ethics committees of the University of Tokyo and Keio University. The participants provided their written informed consent to participate in this study.

## Author Contributions

YY, BY, JH, MS, and MM designed the study. YY, BY, JH, HY, KN, MY, MK, and KK acquired the data. YY, BY, JH, and RU analyzed and interpreted the results of the data. YY, BY, JH, and MM drafted the manuscript. All authors contributed to the article and approved the submitted version.

## Conflict of Interest

The authors declare that the research was conducted in the absence of any commercial or financial relationships that could be construed as a potential conflict of interest.
